# Decayed, Missing, and Filled Teeth Index and Periodontal Health in Osteoporotic Patients Affected by BRONJ: An Observational Study

**DOI:** 10.1155/2013/231289

**Published:** 2013-12-12

**Authors:** Giacomo Oteri, Ennio Bramanti, Valentina Nigrone, Marco Cicciù

**Affiliations:** ^1^Department of Biomedical Sciences and Specialist Medical-Surgical Dentistry, University of Messina, Via Consolare Valeria, 98100 Messina, Italy; ^2^Human Pathology Department, Dental School, University of Messina, Via Consolare Valeria, 98100 Messina, Italy

## Abstract

The aim of this paper is to describe the incidence of decayed, missing, and filled teeth (DMFT) and periodontal disease in 32 osteoporotic patients affected by bisphosphonate-related osteonecrosis of the jaw (BRONJ). Moreover, an investigation between the obtained data and 20 patients treated with bisphosphonate drugs and with no evidence of ONJ has been performed. Osteonecrosis of the jaws is a rare complication in a subset of patients receiving bisphosphonate drugs. Based on a growing number of case reports and institutional reviews, this kind of therapy can cause exposed and necrotic bone specifically in the jawbones. From April 2009 to June 2012, 32 osteoporotic patients treated with oral or intravenous (I.V.) bisphosphonates have been recorded. The patients' oral health has been compared with 20 bisphosphonates patients with no ONJ. The incidence of decayed, missing, and filled teeth (DMFT) and periodontal disease was recorded in all patients and student's *t*-test was applied for comparing the two investigated groups data. Data demonstrated how the poor dental hygiene and periodontal disease of the BRONJ patients' are connected with the occurrence of jawbone necrosis.

## 1. Introduction 

Osteoporosis is a systemic skeletal disorder characterized by skeletal fragility, microarchitectural variation, and low bone mineral density estimated with a T-score for bone mineral density below −2.5 (National Institutes of Health Consensus Conference) [1]. Osteoporosis is one of the most common chronic diseases referred in 1/3 postmenopausal women and 1/5, men over the age of 50 years (European Parliament Osteoporosis Interest Group and EU Osteoporosis Consultation Panel 2004) [2]. Although it is widely recognized that low bone mass is not the only determinant of bone fragility, the strength of the skeleton is influenced by other bone tissue properties, collectively named “bone quality” [3, 4]. Change of bone remodelling pattern in osteoporosis patients resulted in perforation of trabecular plates and loss of cancellous trabecular elements with consequent bone mineral density reduction.

Bisphosphonates are a new class of drugs indicated for use in patients with osteoporosis, Paget's disease of bone, hypercalcemia in a malignant disease, osteolytic bone metastases, and osteolytic lesions of multiple myeloma. Despite the benefits of bisphosphonate therapy like increasing bone density and preventing bone pathological fractures, osteonecrosis of the jaw is a rare complication in a subset of patients receiving these drugs. This complication often occurs after simple dentoalveolar surgery. The pathogenesis for this complication is still debated. It seems to be related to the profound inhibition of osteoclast function and bone remodelling even if it has been documented also in patients not receiving bisphosphonates [2, 4, 5].

The rationale for the use of bisphosphonates in the postmenopausal patient for osteoporosis management is provided by a sequence of modified biological events [2–5]. The introduction of bisphosphonates therapy for the treatment of bone remodelling diseases has been correlated with the increasing of bisphosphonate-related osteonecrosis (BRONJ). During the past two decades, BP therapy has become an elective clinical intervention for osteoporosis. Oral BP therapy was prescribed in 73% of 6.3 million visits for osteoporosis in 2003 and it is estimated that over 190 million prescriptions for oral BP have been dispensed in the world [6, 7]. The enzyme target for BPs is farnesyl pyrophosphate synthase by this enzyme inhibition in the osteoclast; BP interferes with geranylgeranylation (attachment of the lipid to regulatory proteins), thus inducing osteoclast inactivation and also apoptosis [8]. Osteoclast inhibition leads to a reduction in bone turnover and the prevention of bone resorption [9]. The potency of osteoclast inhibition is related to the chemical structure of the BP, with nitrogen-containing BPs (including alendronate, risedronate, ibandronate, and zoledronate) being up to 10,000 times more potent than non-nitrogen-containing BPs [10].

This study tested the hypothesis that osteoporotic patients affected by BRONJ have a poorer dental and periodontal history than non-BRONJ patients, analysing them retrospectively, comparing medical and oral history and standardized radiographs.

## 2. Material and Methods

**Figure 1:**
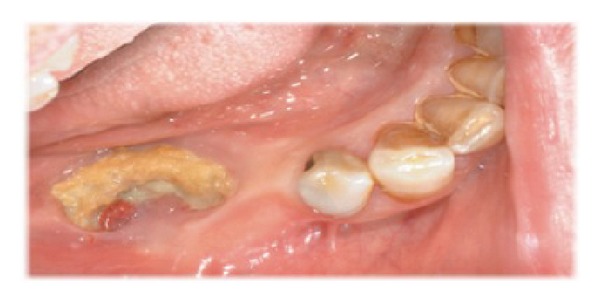
Sample of patients affected by BRONJ.

**Figure 2:**
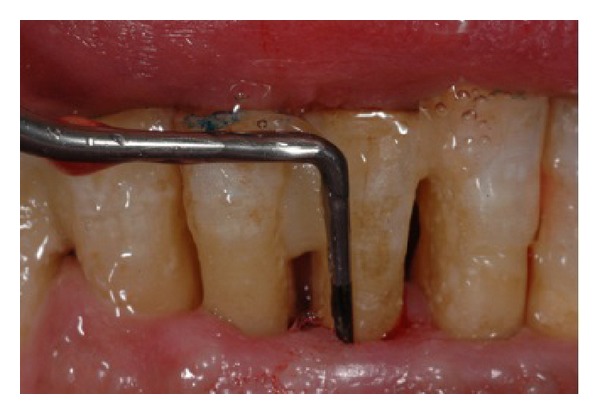
Sample of periodontal probe on osteoporotic patients without BRONJ.

From April 2007 to June 2012, the oral condition of periodontal health and caries prevalence of 32 osteoporotic patients affected by BRONJ (Figure 1) have been recorded (mean age 60.25 years, range 44–80 years); the same condition was reported for 20 osteoporotic patients (mean age 61.95 years, range 44–80 years) without ONJ. The patients were referred to the “A.O.U Gaetano Martino” dental clinic, the Department of Biomedical Sciences, University of Messina, and Specialist Medical-Surgical Dentistry, from the Geriatric Department of the IRCSS Neurolesi Bonino Pulejo Messina. One of the investigators clinically inspected the oral cavity by performing a periodontal evaluation by using the periodontal screening index (PSI) at four proximal sites per tooth. According to the PSI scores, the findings were diagnosed as follows: scores 0–2 “no periodontitis” and scores 3 and 4 “periodontitis” (Figure 2) [11, 12].

Edentulous patients were not included in the study. Clinical investigation was completed by a mouth mirror for evaluating the presence of dental caries and the number of missed and filled teeth.

Patients were classified as affected by BRONJ according to the American Association of Oral and Maxillofacial Surgeons (AAOMS) guidelines. All the patients included in the study were taking BP for osteoporotic therapy. The questionnaire included age, gender, modality of administration, duration and type of BP, associated pathology, and possible corticosteroid usage. Patients were asked if they had undergone any oral surgical procedure since they started bisphosphonate therapy. The same examiner visited all the patients. The data was collected in a database and the oral check-up recorded dental caries, missing teeth, and filled teeth. The data were classified accordingly with the DMFT index, namely, D (decayed), M (missing), and F (filled) teeth for both groups. Digital panoramic radiographs were taken of each patient (Orhophos Plus Ds, Sidexis, with image processor Sidexis Next Generation 1.31; Long Island City, NY). The DMFT index was epidemiologically assessed both from the patient's radiograph and through clinical examination [11]. The PSI was taken with the WHO probe which was inserted into the periodontal pocket in the apical direction parallel to the tooth axis [11, 12]. Every tooth was probed at four sites (mesiovestibular, distovestibular, mesiooral, and distooral) and the PSI score (0 to 4) was recorded. The highest score was determined for each sextant. A periodontal pocket deeper than 4 mm was considered a pathologic periodontal condition according to the Community Periodontal Index of Treatment Needs' assessment sequence. The following classifications were made for each participant in this study: PSI scores 0, 1, and 2: “no periodontitis”; PSR/PSI score 3 and 4: “periodontitis.”

Student's *t*-test, in statistics, is defined as a method of testing hypotheses about the mean of a small sample drawn from a normally distributed population when the population standard deviation is unknown [13]. For this reason the Student's *t*-test was used to statistically analyse the DMFT index and the PSI score in the groups of the investigated patients.

## 3. Results and Discussion

A total of 52 patients have been examined. The study involved two groups of patients: A Control Group, 20 osteoporotic patients (16 females, 4 males) without BRONJ (Table 1); B Group, 32 osteoporotic patients (26 females, 6 males) affected by BRONJ (Table 2).

The mean age of BRONJ patients was comparable and similar to the Control Group.

**Figure 3:**
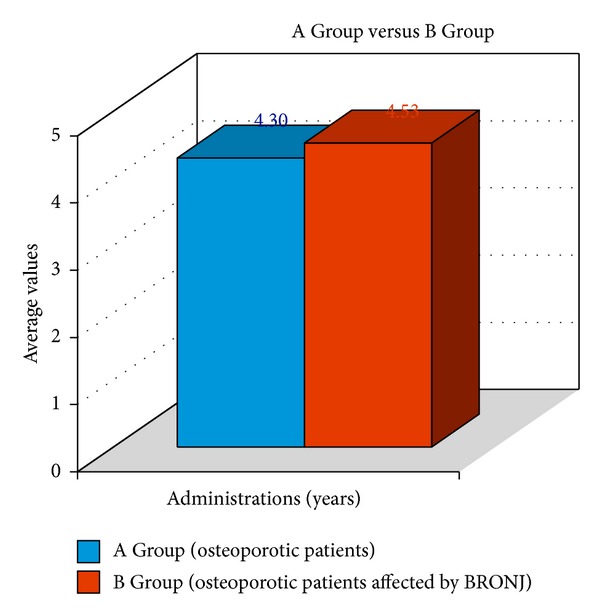
Years of BP administration—A and B Groups.

This was also true for bisphosphonates administration duration that is comparable between the two groups (Control: 4.3 years ± 2.5 years; BRONJ: 4.5 years ± 2.6 years) and the *t* value did not show any statistical difference in A and B Groups (*P* = 0.7503) (Figure 3).

*D: Number of Decayed Teeth*

**Figure 4:**
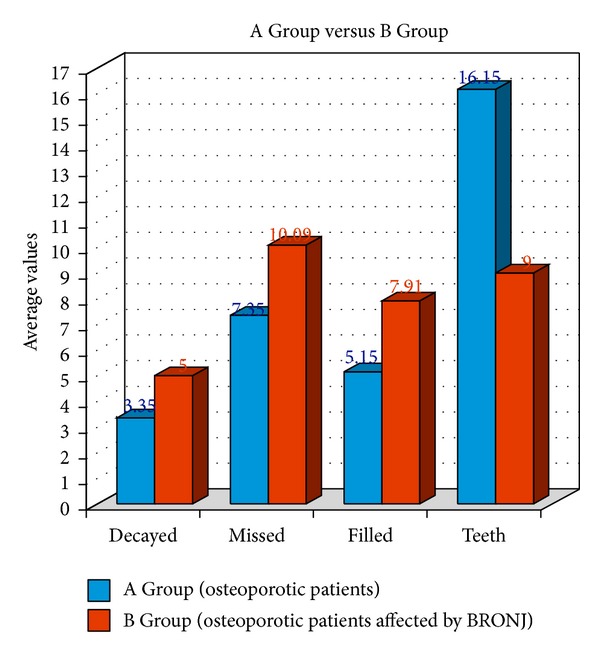
Average number of decayed, missed, and filled teeth recorded for patients in each group.

- The number of untreated caries per patient ranged from 0 to 6 in the Control Group with an average of 3.3 ± 1.5 decayed teeth per person (Table 1 and Figure 4).
- The number of untreated caries per patient ranged from 2 to 10 in the BRONJ Group with an average of 5 ± 1.9 decayed teeth per person (Table 2 and Figure 4).

*M: Number of Missed Teeth.* According to the International Consensus, a complete denture is composed of 28 teeth in the upper and lower jaws, avoiding the presence of the wisdom teeth [14].

- The number of missed teeth per patient ranged from 2 to 18 in the Control Group with an average of 7.3 ± 3.9 missed teeth per person (Table 1 and Figure 4).
- The number of missed teeth per patient ranged from 4 to 18 in the BRONJ Group with an average of 10.0 ± 3.8 missed teeth per person (Table 2 and Figure 4).

*F: Number of Filled Teeth*

- The number of filled teeth per patient ranged from 1 to 9 in the Control Group with an average value of 5.1 ± 2.2 teeth (Table 1 and Figure 4).
- The number of filled teeth per patient ranged from 1 to 16 in the BRONJ Group with an average value of 7.9 ± 3.3 teeth (Table 2 and Figure 4).

*T: Number of Healthy Teeth*

- The number of healthy teeth remaining per patient ranged from 10 to 24 in the Control Group with an average value of 16.1 ± 3.8 teeth (Table 1 and Figure 4).
- The number of healthy teeth per patient ranged from 3 to 14 in the BRONJ Group with an average value of 9 ± 3.4 teeth (Table 2 and Figure 4).

*Probing Pocket Depth*

**Figure 5:**
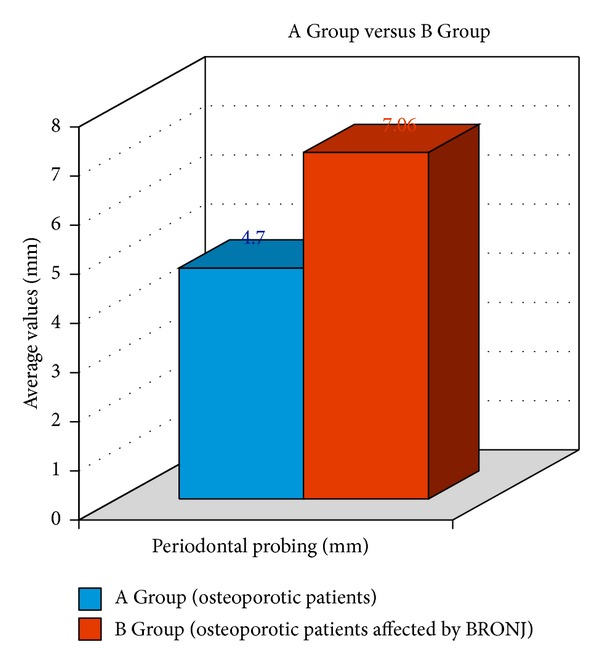
Periodontal probing average depth recorded for patients in each group.

- The periodontal pockets per patient ranged from 1 to 9 mm of depth in the Control Group with an average of 4.7 ± 2.3 mm (Table 1 and Figure 5).
- The periodontal pockets per patient ranged from 4 to 10 mm of depth in the BRONJ Group with an average of 7.0 ± 1.8 mm (deep pocket and severe periodontal disease) (Table 2 and Figure 5).

All of the 32 BRONJ patients of the B Group present a larger and meaningful number of decayed (*P* = .001342731), missed (*P* = .018622821), and filled (*P* = .000901775) teeth (Table 3).

This study shows that the oral health of a consecutive group of BRONJ patients for an oral health check and treatment, generally, had a poorer standard of oral health than bisphosphonate patients with no evidence of ONJ. We tested the hypothesis that the poor periodontal conditions of osteoporotic patients might increase susceptibility to BRONJ.

The American Academy of Oral and Maxillofacial Surgeons defined BRONJ as the presence of necrotic bone in the oral cavity for at least 8 weeks in a patient who is taking (or has taken) BP and who has not received radiation to the head and neck. Four different grades (from 0 to III) of pathology based on clinical severity of symptoms have been identified [15]. Patients affected by BRONJ may have swelling of oral and perioral tissues, pain, bleeding, persistent purulent discharge and draining fistulas, severe halitosis, lower lip paraesthesia, and mobility and loosening of teeth. Patients receiving bisphosphonates who are undergoing dentoalveolar surgery (extractions, dental implant placement, periapical surgery, and periodontal surgery involving osseous injury) are seven times more likely to develop BRONJ than patients who are not having dental surgical procedures [16]. Osteonecrosis of the jaw with oral BP formulations has an estimated incidence of less than one case per 100,000 person-years of exposure. Patients under treatment with oral bisphosphonate therapy have a considerably lower risk of BRONJ than patients treated with IV bisphosphonates. Based on data from the manufacturer of alendronate (Merck), the incidence of BRONJ was calculated to be 0.7/100,000 person/years of exposure. Correspondence with Alastair Goss, DDSc (September 2006), reported that the estimated incidence of BRONJ for patients treated weekly with alendronate is 0.01–0.04%, based on prescription data in Australia. Following extractions, this rate increased to 0.09–0.34%. Other studies pointed out how the incidence of BRONJ in osteoporosis patients taking oral BPs could be significantly higher than previously reported [17]. It has been suggested that BRONJ can be predicted with a combination of environmental and genetic risk factors. Genetic risk factors include polymorphisms of the CYP2C8 gene [18], vascular endothelial growth factor polymorphisms, and mutations in the prothrombin gene [19, 20]. A published case series including at least 10 patients identified in a single city or a limited geographical region showed that 55% of reports come from Mediterranean countries such as Italy (25, 24, and 13 cases; total = 62) [18–21].

While other broader studies on dental and periodontal conditions of patients on bisphosphonates with and without BRONJ including clinical data on the activity and progression of oral diseases might disclose differences that did not reach a statistically significant difference [21], our experimental study found a highly statistically significant difference both of DMFT index and of periodontal probing depth (Table 3).

The incidence of periodontal disease was statistically significant (*P* = .000452719) (Table 3). Furthermore all the BRONJ patients have a pathological periodontal pocket of 4 mm at least (Table 2). The limits of the confidence interval are 6.3 mm and 7.7 mm on average of the probing depth within which there is the a probability of 95% to find the “true” periodontal probing average of BRONJ patients.

Prevention by the combination of improving oral health and improved management of their bone disease is the best management of these cases [22–26].

## 4. Conclusions

This study also shows the fairly simple treatment measures we required for making the patients' oral cavities healthy. Commonly, this involved extraction of hopeless teeth, endodontics and referral back to their general dentist for simple scaling, and cleaning and oral hygiene instruction.

When extractions were performed the patients had a full informed consent discussion. Extractions should be performed in accordance with the recommendations in the Therapeutic Guidelines, namely, preextraction antibiotic cover, minimal trauma, and suturing of the socket avoiding postoperative complications like bleeding or pain. All the patients were checked after one week and one month. Although some patients had a delayed healing, none developed ONJ [24, 26]. Given that the risk of ONJ for patients on oral bisphosphonates has been calculated in the range of 1 in 225 to 1 in 1100, then the probability of such a study being completed at a single centre is low.

Data results clearly showed how the frequencies of decayed, missed, and filled teeth and periodontal disease were significantly higher in the BRONJ patients group than in the Control Group (Table 3 and Figures 4 and 5).

For this reason, clinicians should recommend patients with osteoporosis paying special attention to the maintenance of their oral hygiene. Regular dental visits are fundamental, with frequent scaling and root planning and conservative, periodontal, and endodontic therapy as needed. Dental extractions should be performed only in the case of hopeless teeth.

The severity of oral health increases with excessive duration of administration of the drug. Black et al. showed that prolonged use of bisphosphonates over five years does not have a therapeutic value because the risk of vertebral fracture remains similar and mineral bone density does not tend to improve [27]. New clinical therapeutic options like growth factor application may be applied for reducing patient pain and for improving the clinical healing after jawbone necrosis [28].

Patients with BRONJ appear to have a higher incidence of periodontal disease and for this reason they should undergo supervised dental care in order to maintain sufficient periodontal attachment without further disease progression. This study highlights how patients with existing untreated periodontal disease and a higher DMFT Index undergoing BP therapy may be at a higher risk for BRONJ and need close supervision and care of their dental condition (Table 3).

## Figures and Tables

**Table 1:** Anamnestic and clinical details of A Group—20 osteoporotic non-BRONJ patients.

Age	OS type	BP therapy	BP (years)	Associated pathology	Corticosteroid assumption	D	M	F	Periodontal probing (mm)
52	1	Ale* + Clo**	2	No	No	4	6	6	3
67	1	Clo** + Ris***	3	Diabetes mellitus II	No	6	8	5	2
59	2	Clo**	5	No	Yes	3	4	7	5
73	1	Ale*	6	No	No	3	11	2	4
77	1	Ale*	8	Rheumatoid arthritis	No	5	9	5	7
44	1	Iba****	2	No	No	2	3	4	3
61	1	Ale*	3	Rheumatoid arthritis	No	4	5	8	4
70	1	Clo** + Ris*** + Ner*****	9	Hypertension	No	4	10	6	6
49	1	Ale* + Clo**	4	No	No	3	5	3	2
55	1	Ner*****	3	No	No	4	6	8	7
78	2	Ale*	6	Rheumatoid arthritis	Yes	6	12	4	9
65	1	Ale* + Clo**	3	No	No	5	7	1	3
52	1	Ris***	2	No	No	3	4	7	6
80	1	Ale*	10	Cardiovascular diseases	No	2	18	2	9
66	2	Ale* + Clo**	4	Diabetes mellitus II	Yes	1	6	9	2
54	1	Clo** + Ris*** + Ner*****	4	No	No	3	5	8	4
47	1	Ale* + Clo**	2	No	No	0	2	6	1
71	1	Iba****	6	Diabetes mellitus II	No	2	14	3	6
59	2	Ale*	1	No	Yes	4	6	5	5
60	1	Ale* + Clo**	3	Hypertension	No	3	6	4	6

(i) Ale: alendronate: oral assumption, **Clo: clodronate: oral assumption, ***Ris: risedronate: oral assumption, ****Iba: ibandronate: oral assumption, and *****Ner: neridronate: IV assumption.

(ii) OS type: postmenopausal ostep “1”; corticosteroid related osteop “2.”

**Table 2:** Anamnestic and clinical details of B Group—32 osteoporotic BRONJ patients.

Age	OS type	Trigger event	Place of infection	BRONJ stage	BF therapy	BF (years)	Associated pathology	Cortc ST	D	M	F	Period probing (mm)
66	1	Teeth extract	MAN	2	Ale*	6	Diabetes mellitus II	No	4	12	10	7
60	1	Teeth extract	MAN	1	Ale*	5	Diabetes mellitus II	No	6	8	7	6
56	1	Cyst enucl	MAN	1	Clo**	4	No	No	10	5	6	5
72	1	Teeth extract	MAS	1	Ale* + Clo**	8	No	No	4	18	6	7
80	1	Teeth extract	MAS	1	Ale*	11	No	No	5	17	1	9
61	2	Teeth extract	MAS	3	Ale* + Clo**	1	Rheumatoid arthritis	Yes	3	11	12	8
63	1	Teeth extract	MAN	2	Ale*	5	Hypertension	No	4	14	7	9
47	1	Teeth extract	MAS	1	Ale*	3	No	No	10	6	3	10
51	1	Periodontal infection	MAN	1	Ale* + Clo**	4	No	No	8	4	9	6
56	1	Teeth extract	MAN	0	Ale*	5	Rheumatoid arthritis	No	3	10	9	9
49	1	Teeth extract	MAN	3	Ris***	2	No	No	4	17	4	6
47	1	Implant insertion	MAS	1	Ner*****	3	No	No	8	4	6	4
61	1	Teeth extract	MAS	1	Ale*	6	No	No	6	11	3	8
51	1	Teeth extract	MAN	1	Ale*	3	No	No	5	8	10	5
63	1	Implant removal	MAN	1	Ale* + Clo**	4	No	No	4	9	10	7
62	1	Teeth extract	MAN	2	Ale* + Clo**	5	No	No	5	10	9	9
69	1	Periodontal infection	MAN	2	Ale* + Clo**	6	Cardiovascular diseases	No	5	14	9	5
75	1	Not identified	MAS	1	Ale*	9	Diabetes mellitus II	No	6	11	10	9
77	1	Teeth extract	MAN	1	Ale*	11	Rheumatoid arthritis	No	5	17	6	8
74	1	Teeth extract	MAS	1	Clo**	8	Hypertension	No	4	9	5	10
65	2	Teeth extract	MAN	1	Clo** + Ris	4	Rheumatoid arthritis	Yes	3	10	16	9
67	2	Teeth extract	MAN	2	Ale*	2	Rheumatoid arthritis	Yes	4	6	14	7
54	2	Implant insertion	MAS	1	Clo** + Ris*** + Ner*****	3	No	Yes	4	8	11	5
64	1	Teeth extract	MAN	0	Ale*	4	Hypertension	No	7	6	8	9
59	1	Periodontal infection	MAN	0	Ale*	2	No	No	6	12	4	4
56	1	Teeth extract	MAN	0	Iba****	4	No	No	3	10	7	6
63	1	Teeth extract	MAS	0	Iba****	3	Cardiovascular diseases	No	4	9	6	7
47	2	Teeth extract	MAS	1	Ale*	2	No	Yes	6	6	9	6
48	1	Teeth extract	MAN	1	Ale*	1	No	No	2	9	8	5
44	1	Implant insertion	MAN	1	Ale* + Clo**	2	No	No	5	8	5	4
56	1	Teeth extract	MAS	1	Ale* + Clo**	3	No	No	4	15	10	9
65	2	Teeth extract	MAN	2	Ale*	6	Rheumatoid arthritis	Yes	3	9	13	8

(i) Ale: alendronate: oral assumption, **Clo: clodronate: oral assumption, ***Ris: risedronate: oral assumption, ****Iba: ibandronate: oral assumption, and *****Ner: neridronate: IV assumption.

(ii) OS type: postMenopausal ostep “1”; Corticosteroid related osteop “2.”

**Table 3:** A Group versus B Group—evaluation of clinical and statistical significance.

	Administrations (years)	D	M	F	Periodontal probing (mm)
A Group (osteoporotic patients)	4.3 (2.49)	3.35 (1.53)	7.35 (3.96)	5.15 (2.28)	4.7 (±2.29)
B Group (BRONJ patients)	4.53 (2.6)	5 (1.93)	10.09 (3.85)	7.91 (3.34)	7.06 (±1.85)
*P* value	0.75	0.001342731*	0.018622821*	0.000901775*	0.000452719*

Average values ± SD; DMFT index; *statistically significant difference.
